# Supplementary material for: Pseudo-Hydrogen Passivation: A Novel Way to Calculate Absolute Surface Energy of Zinc Blende (111)/(¯1 ¯1 ¯1) Surface
Source: Sci Rep. 2016 Feb 1;6:20055. doi: 10.1038/srep20055 (PMC4810009; doi:10.1038/srep20055)
Supplement: Supplementary Information [file srep20055-s1.pdf]

Supplemental material: Pseudo-Hydrogen Passivation_A Novel Way to Calculate Absolute Surface Energy of Zinc Blende (111)/($\overline{1}\overline{1}\overline{1}$) Surface

Yiou Zhang, Jingzhao Zhang, Kinfai Tse, Lun Wong, Chunkai Chan, Bei Deng and Junyi Zhu

Department of Physics, the Chinese University of Hong Kong, Hong Kong

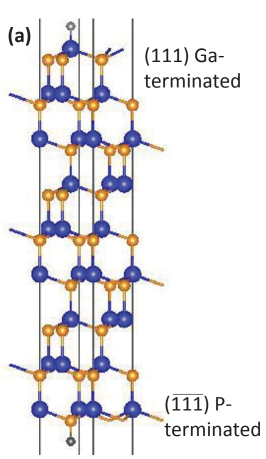

Fig. S1.(Color online) Illustration of slabs used in the calculation, taking GaP as example. Slab along [111] direction is shown in (a) while slab along [110] direction is shown in (b). Blue ball represents Ga and orange ball represents P. Grey ball attached to surface atoms indicates corresponding pseudo-H atom.

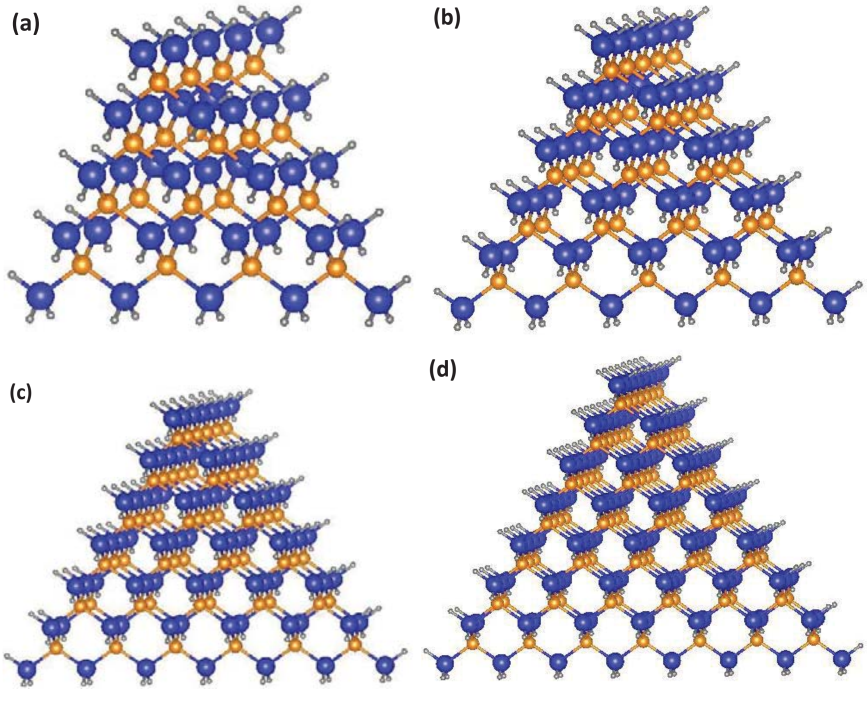

Fig. S2 Illustration of tetrahedral clusters, with size (a) n=5, (b) n=6, (c) n=7, and (d) n=8, taking GaP as an example. Blue ball represents Ga and orange ball represents P, whereas small grey ball represents pseudo-H atom.
